# Incidence, Risk Factors, and Outcomes Associated With In-Hospital Acute Myocardial Infarction

**DOI:** 10.1001/jamanetworkopen.2018.7348

**Published:** 2019-01-18

**Authors:** Steven M. Bradley, Joleen A. Borgerding, G. Blake Wood, Charles Maynard, Stephan D. Fihn

**Affiliations:** 1 Minneapolis Heart Institute, Minneapolis, Minnesota; 2 Minneapolis Heart Institute Foundation, Minneapolis, Minnesota; 3 Veterans Affairs Puget Sound Health Care System, Seattle, Washington; 4 Department of Medicine, University of Washington, Seattle

## Abstract

**Importance:**

Studies of acute myocardial infarction (AMI) occurring outside the hospital have informed approaches to addressing risk, treatment, and patient outcomes. Similar insights for in-hospital AMI are lacking.

**Objective:**

To determine the incidence, risk factors, and outcomes associated with in-hospital AMI.

**Design, Setting, and Participants:**

Cohort, nested case-control, and matched cohort study of patients hospitalized in US Veterans Health Administration facilities between July 2007 and September 2009. The incidence of in-hospital AMI was determined from a complete cohort of in-hospital AMI relative to the total number of inpatient admissions. From the in-hospital AMI cohort, detailed medical record review was performed on 687 cases and 687 individually matched controls. Risk factors and outcomes associated with in-hospital AMI were determined from matched comparison of in-hospital AMI cases to hospitalized controls.

**Exposures:**

Candidate risk factors for in-hospital AMI included characteristics at the time of admission and in-hospital variables prior to the index date.

**Main Outcomes and Measures:**

In the determination of the incidence and risk factors associated with in-hospital AMI, the outcome of interest was in-hospital AMI. All-cause mortality was the main outcome of interest following in-hospital AMI.

**Results:**

A total of 5556 patients with in-hospital AMI (mean [SD] age, 73 [10] years; 5456 [98.2%] male) were identified among 1.3 million admissions, with an incidence of 4.27 in-hospital AMI events per 1000 admissions. Independent risk factors associated with in-hospital AMI included intensive care unit setting, history of coronary artery disease, heart rate greater than 100 beats/min, hemoglobin level less than 8 g/dL, and white blood cell count 14 000/μL or greater. Compared with the matched control group, mortality was significantly higher for patients with in-hospital AMI (in-hospital mortality, 26.4% vs 4.2%; 30-day mortality, 33.0% vs 10.0%; 1-year mortality, 59.2% vs 34.4%).

**Conclusions and Relevance:**

In-hospital AMI was common and associated with common cardiovascular risk factors and markers of acute illness. Patient outcomes following in-hospital AMI were poor, with 1-year mortality approaching 60%. Further study of in-hospital AMI may yield opportunities to reduce in-hospital AMI risk and improve patient outcomes.

## Key Points

**Question:**

What are the incidence, risk factors, and outcomes associated with in-hospital acute myocardial infarction (AMI)?

**Findings:**

This cohort study of 1.3 million patients hospitalized in US Veterans Health Administration facilities found an incidence of in-hospital AMI of 4.27 per 1000 admissions, and risk factors associated with in-hospital AMI included history of coronary artery disease, elevated heart rate, low hemoglobin level, and elevated white blood cell count. Compared with a matched control group, mortality was significantly higher for in-hospital AMI.

**Meaning:**

In-hospital AMI is common and is associated with prior cardiovascular disease, physiological disturbances, and poor survival.

## Teaser

This cohort study investigates the incidence, risk factors, and outcomes associated with in-hospital acute myocardial infarction (AMI) among a population of patients hospitalized in US Veterans Health Administration facilities.

## Introduction

Most studies of acute myocardial infarction (AMI) epidemiology and treatment have focused on patients who experience the onset of AMI outside of the hospital. Insights from these studies have informed risk factors and optimal treatment of AMI, which have led to subsequent reductions in AMI incidence and mortality.^1,2^ It is increasingly recognized that AMI also occurs among patients already hospitalized for other conditions.^3,4^ Insights on AMI occurring during hospitalization are limited.

Patients with in-hospital ST-segment elevation myocardial infarction (STEMI) have been shown to experience delays in revascularization and worse short-term outcomes when compared with patients experiencing STEMI onset in the outpatient setting.^5,6,7,8^ Although these comparisons have identified potential treatment gaps in the care of in-hospital STEMI,^9^ less is known about the patient characteristics and long-term outcomes associated with in-hospital AMI. Additionally, prior studies have compared in-hospital AMI outcomes with those of individuals with outpatient onset AMI who survive to hospital admission, potentially biasing comparisons of patient characteristics and outcomes.^3,4,5,6,7,8,10,11^ Finally, STEMI reflects less than 25% of all myocardial infarctions^2^ and few studies of in-hospital AMI have included non–ST-segment elevation myocardial infarctions (NSTEMI).^3,4,11,12,13^ Little is known about the full spectrum of in-hospital AMI.

The objectives of this study were to describe the incidence, risk factors, and long-term mortality outcomes associated with in-hospital AMI. We determined the incidence of in-hospital AMI in the Veterans Administration (VA) health care system using data from the Veterans Health Administration (VHA) External Peer Review Program (EPRP), which includes a 100% sample of all AMIs. Reports of the differences between patients with in-hospital AMI and similar patients who were admitted to the hospital but did not develop AMI are limited.^12^ With this is in mind, we used a case-control design to identify risk factors associated with developing in-hospital AMI and to compare subsequent 1-year mortality rates.

## Methods

### Data Source, Patient Population, and Outcomes for In-Hospital AMI Incidence

To determine the incidence of in-hospital AMI, we identified patients with in-hospital AMI from data collected as part of the VHA EPRP for quality monitoring and improvement. This program captures information for a variety of medical conditions and procedures, including all hospitalized patients with AMI according to *International Classification of Diseases, Ninth Revision* (*ICD-9*), diagnosis codes (410.xx). Working with the EPRP abstraction contractor, West Virginia Medical Institute, Charleston, the VHA Office of Quality and Performance generated a list of all patients with *ICD-9* diagnosis codes of 410.xx from administrative data housed at the Austin Automation Center in Austin, Texas. This patient list was transmitted to VHA hospitals, where both paper and electronic medical records were manually abstracted by trained abstractors using standard reporting forms. Abstracted data were then entered into a database maintained by the contractor. The total number of inpatient admissions in the VHA for the period of study was determined from in-patient bed section files on the Austin mainframe. This study followed the Strengthening the Reporting of Observational Studies in Epidemiology (STROBE) reporting guideline for observational studies. This study was approved by the VA Puget Sound Human Research Protection Program and waiver of informed consent was granted.

We identified patients with in-hospital AMI, defined as AMI diagnosis more than 24 hours after admission to the same hospital based on coding by the EPRP abstractor, occurring between July 2007 and September 2009.

### Data Source and Patient Population for In-Hospital AMI Risk Factors and Outcomes

We performed a nested case-control and matched cohort study of cases and controls to determine risk factors and outcomes associated with in-hospital AMI. We identified cases from the cohort of patients with in-hospital AMI identified by the VHA EPRP. Information on patient controls for comparison with in-hospital AMI cases was obtained from in-patient bed section files on the Austin mainframe.

### Selection of In-Hospital AMI Cases

To permit comparison with patients in the control group and provide increased specificity in case definition, cases included patients with in-hospital AMI who were aged 50 years or older at the time of the event and admitted to a medical bed service with a diagnosis other than ischemic heart disease by *ICD-9* diagnosis codes (410-414). Classification as to whether AMI occurred in-hospital was initially based on coding by the EPRP abstractor and the admitting diagnosis from VHA bed section files. The final determination was made by study abstractors who reviewed the electronic health record for criteria of AMI in accordance with universal criteria.^14^ These criteria included a typical increase and decrease of cardiac-specific enzyme levels plus 1 of the following: typical ischemic symptoms (chest pain or dyspnea); electrocardiographic changes indicative of ischemia (ST-segment elevation or depression or development of new pathologic Q waves); and/or evidence of a significant coronary lesion (≥50% in the left main coronary or ≥70% in any other major epicardial coronary) on coronary angiography. For each case, the timing of AMI was determined by the date and time of the first elevated troponin value or first electrocardiogram automated interpretation showing electrocardiographic changes indicative of ischemia, whichever occurred first. As troponin assays and reference ranges vary by facility, an elevated troponin level consistent with AMI was defined in accordance with the local facility assay and reference range. Patients with AMI onset within 24 hours of admission were excluded to ensure cases represented AMIs that truly occurred in-hospital. Characterization of STEMI and NSTEMI was on the basis of *ICD-9* coding without additional medical record review as prior work suggested our study would have inadequate sample size for stratified analysis on this characteristic.^3^ Accordingly, analysis by STEMI vs NSTEMI was not planned beyond descriptive findings.

Two additional exclusion criteria were applied. First, patients who were transferred to VHA hospitals from outside medical facilities were excluded because key baseline variables were not available. Second, we excluded patients with perioperative AMI as defined by admission to a surgical bed unit or a surgical procedure within 72 hours of the event, given the extensive literature, risk indices, and practice guidelines that exist for the perioperative AMI population.

### Selection of the Control Group

The control group included patients aged 50 years or older who were admitted to a medical bed section with a diagnosis other than ischemic heart disease (*ICD-9* diagnosis codes 410-414) and did not receive a diagnosis of AMI at any time during their hospitalization. Patients who were transferred in from outside hospitals, admitted to surgical bed sections, or hospitalized for less than 3 days were excluded. In addition, we excluded individuals without a diagnosis of AMI during the hospitalization despite evidence of troponin elevation that was identified in medical record review. The day after admission was used as the enrollment date for the control group. Using VHA bed section files, we constructed a sampling frame for control patients; cells for sampling were constructed according to hospital, 10-year age categories, and 6-day admission categories. Within each cell, 1 control individual was randomly selected, such that in comparison with the case patient, he or she was from the same hospital, was within 5 years of age, and was admitted within 90 days. The electronic medical records of control individuals were reviewed by trained study abstractors to ensure there was no occurrence of AMI during the admission.

### Candidate Risk Factors and Covariates for Risk-Adjusted Outcomes

Candidate risk factor variables for in-hospital AMI included patient characteristics at the time of admission and in-hospital variables prior to the index date. Patient characteristics present at the time of admission that were assessed included demographic characteristics (age, sex, and race), medical history, and clinical risk factors (hypertension, dyslipidemia, current tobacco use, coronary artery disease, prior myocardial infarction, prior percutaneous coronary intervention, prior coronary artery bypass graft surgery, heart failure [prior congestive heart failure or cardiomyopathy], cerebrovascular disease, peripheral vascular disease, diabetes mellitus, chronic obstructive pulmonary disease, obstructive sleep apnea, chronic kidney disease, chronic liver disease, anemia, depression, alcohol dependence or abuse, posttraumatic stress disorder, dementia, or malignancy), which were determined from review of the medical history in the medical record on admission. In-hospital variables included bed section at the time of event (medical, intensive care unit, or other), the most recent vital signs within 24 hours of the event (body mass index, heart rate, and systolic blood pressure), and the most recent laboratory values within 48 hours of the enrollment date (day of AMI for case patients; day after admission for control individuals) for in-hospital laboratory testing that was performed in more than 90% of case and control participants (white blood cell count, hemoglobin level, platelet count, and levels of potassium, sodium, serum urea nitrogen, and creatinine). All candidate risk factors were subsequently used as covariates in risk-adjusted outcome models. There were no missing data on covariates with the exception of laboratory testing (<1%) and vital sign data (body mass index, 6.3%; heart rate, 5.1%; and systolic blood pressure, 5.1%). Stochastic regression methods were used to impute missing laboratory and vital sign data.

### Validation of Medical Record Abstraction

Full-time medical record abstractors were trained on 10 sample cases and their results were compared and discussed in preparatory study meetings to resolve discrepancies and develop and revise an abstracting algorithm. After completion of this phase, each abstractor independently screened a test data set consisting of 50 potential cases (37 eligible and 13 ineligible) and 50 potential controls (40 eligible and 10 ineligible). The abstractors demonstrated 100% agreement on both eligibility and the reasons for ineligibility (eTable 1 in the Supplement).

The second phase of abstraction validation focused on aspects of admission data, vitals, laboratories, medications, discharge diagnoses, and follow-up. Agreement was greater than 90% for most data elements (eTable 2 in the Supplement). The final phase of abstraction validation related to hospital events, procedures, and cardiac catheterization laboratory procedures. The agreement on the presence or absence of events was greater than 90% (eTable 3 in the Supplement).

### In-Hospital AMI Risk Factors and Outcomes

The outcome was abstractor-validated in-hospital AMI for our case-control study of risk factors associated with in-hospital AMI. The primary outcome for our matched cohort study of outcomes following in-hospital AMI was all-cause mortality occurring in the year following the date of the index event. Secondary outcomes included in-hospital mortality, 30-day mortality, 1-year all-cause readmission, and admission for AMI. Death was ascertained from the VA Information Resource Center Vital Status File, which compiles data from the Beneficiary Identification Records Locator Subsystem Death file, VA Medicare Vital Status File, and the Social Security Administration Death Master File. In the period following discharge, we identified 1-year all-cause readmission and admission for AMI from VA administrative inpatient data records and *ICD-9* codes for AMI (410.xx).

### Statistical Analysis

To determine the incidence of in-hospital AMI, we report the number of in-hospital AMIs as identified by EPRP relative to the total number of inpatient admissions in the VHA for the period of study.

In the comparison of cases and controls, we first compared patient characteristics at admission and in-hospital variables prior to the event using the χ^2^ statistic for categorical variables and the 2-sample *t* test for continuous variables. We then sought to identify risk factors associated with in-hospital AMI using conditional logistic regression. Given the large number of potential risk factors under consideration, we used sequential model building and retained variables at each step that maintained statistical significance at the 0.10 level. In our first model, we included patient demographic characteristics, past medical history and clinical risk factors, and bed section details. We then added covariates for vital signs in a second model and covariates for laboratory values in a third model. A fourth model was constructed consisting of variables significant at the 0.10 level in any of the 3 prior models.

To compare outcomes between in-hospital AMI cases and controls, we report in-hospital mortality, 30-day mortality, and 1-year mortality rates following the index event for the matched cohort. Kaplan-Meier estimates of the survival function were calculated with cases stratified by STEMI vs NSTEMI designation. We then assessed the unadjusted association between in-hospital AMI and 1-year outcomes from a null multiple logistic regression model. We then included all covariates described above in a fully adjusted model. These models were repeated for the outcomes of in-hospital mortality, 30-day mortality, and 1-year all-cause readmission. Events were too few to permit risk-adjusted models for the outcome of AMI-specific readmission.

All tests for statistical significance were 2-tailed and *P* < .05 was considered statistically significant. All statistical analyses were performed using SAS statistical software version 9.4 (SAS Institute Inc).

## Results

We identified 5556 patients (mean [SD] age, 73 [10] years; 5456 [98.2%] male) with in-hospital AMI, as defined by an AMI diagnosis more than 24 hours after admission to the same hospital based on coding by the EPRP abstractor, which occurred between July 2007 and September 2009. Data on race/ethnicity were not available from EPRP. This represents an incidence of 4.27 AMI events per 1000 admissions based on 1.3 million admissions to VA hospitals during this period.

**Figure 1.**
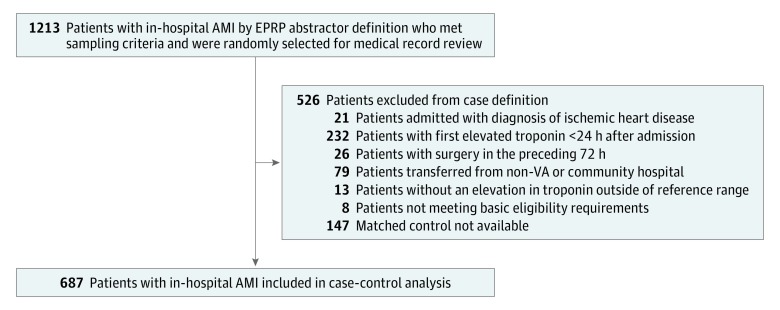
Selection of In-Hospital Acute Myocardial Infarction (AMI) Cases EPRP indicates External Peer Review Program; VA, Veterans Affairs.

Of the 5556 patients identified with in-hospital AMI by EPRP, 1213 patients met sampling criteria and were randomly selected for IHAMI medical record review. Subsequent application of case criteria identified 834 total cases of in-hospital AMI for medical record review, with 687 cases of in-hospital AMI matched to control participants (Figure 1) with *ICD-9* codes designating 587 (85.4%) as NSTEMI and 100 (14.6%) as STEMI. Comparison of case and control patient demographic characteristics, history and clinical risk factors, admission bed type, and pre-event vital signs and laboratory values are shown in Table 1. Case patients had a mean (SD) age of 73.3 (10.1) years and control individuals had a mean (SD) age of 73.4 (10.3) years. Compared with controls, in-hospital AMI cases were significantly more likely to occur in intensive care unit settings (27.1% vs 9.5%) and to have a history of atherosclerotic disease (ie, myocardial infarction, percutaneous coronary intervention, coronary artery bypass graft, cerebrovascular disease, or peripheral vascular disease). Coronary disease risk factors of hypertension, hyperlipidemia, and diabetes were also more common in patients with in-hospital AMI. Laboratory data demonstrated that moderate to severe anemia was more common in patients with in-hospital AMI compared with the control group (68.8% vs 44.8%).

**Table 1.** Patient Characteristics on Admission and In-Hospital Variables Prior to Event for Matched In-Hospital Acute Myocardial Infarction Cases and Controls

Characteristic	No. (%)			*P* Value
	Total (N = 1374)	Cases (n = 687)	Controls (n = 687)	
Age, mean (SD), y	73.3 (10.2)	73.3 (10.1)	73.4 (10.3)	.80
Male	1343 (97.7)	677 (98.5)	666 (96.9)	.05
White race/ethnicity	1073 (78.1)	546 (79.5)	527 (76.7)	.22
Married	666 (48.5)	356 (51.8)	310 (45.1)	.01
Location				
Intensive care unit	251 (18.3)	186 (27.1)	65 (9.5)	<.001
Medical bed	1026 (74.7)	446 (64.9)	580 (84.4)	
Other	97 (7.1)	55 (8.0)	42 (6.1)	
Comorbidities and risk factors				
Hypertension	990 (72.1)	513 (74.7)	477 (69.4)	.03
Hyperlipidemia	759 (55.2)	400 (58.2)	359 (52.3)	.03
Tobacco use	331 (24.1)	172 (25.0)	159 (23.1)	.41
Coronary artery disease	627 (45.6)	372 (54.1)	255 (37.1)	<.001
Prior myocardial infarction	244 (17.8)	164 (23.9)	80 (11.6)	<.001
Prior percutaneous coronary intervention	187 (13.6)	120 (17.5)	67 (9.8)	<.001
Prior coronary artery bypass graft	260 (18.9)	163 (23.7)	97 (14.1)	<.001
Heart failure	374 (27.2)	213 (31.0)	161 (23.4)	.002
Cerebrovascular disease	349 (25.4)	207 (30.1)	142 (20.7)	<.001
Peripheral vascular disease	241 (17.5)	163 (23.7)	78 (11.4)	<.001
Atrial fibrillation	217 (15.8)	96 (14.0)	121 (17.6)	.06
Diabetes	550 (40.0)	299 (43.5)	251 (36.5)	.008
Chronic obstructive pulmonary disease	444 (32.3)	231 (33.6)	213 (31.0)	.30
Obstructive sleep apnea	127 (9.2)	64 (9.3)	63 (9.2)	.93
Chronic kidney disease	397 (28.9)	227 (33.0)	170 (24.7)	.001
Liver disease	122 (8.9)	54 (7.9)	68 (9.9)	.18
Anemia	343 (25)	162 (23.6)	181 (26.3)	.24
Depression	349 (25.4)	156 (22.7)	193 (28.1)	.02
Alcohol dependence or abuse	294 (21.4)	142 (20.7)	152 (22.1)	.51
Posttraumatic stress disorder	102 (7.4)	52 (7.6)	50 (7.3)	.84
Dementia	200 (14.6)	84 (12.2)	116 (16.9)	.01
Malignant neoplasm	443 (32.2)	216 (31.4)	227 (33.0)	.53
Coagulopathy	6 (0.4)	3 (0.4)	3 (0.4)	1.00
Fluid or electrolyte disorder	32 (2.3)	14 (2.0)	18 (2.6)	.47
Gastrointestinal bleed	105 (7.6)	55 (8.0)	50 (7.3)	.61
Presenting characteristics				
Body mass index^a^				
Underweight (<18.5)	115 (8.4)	53 (7.7)	62 (9.0)	.58
Normal (18.5 to <25)	470 (34.2)	246 (35.8)	224 (32.6)	
Overweight (25 to <30)	396 (28.8)	195 (28.4)	201 (29.3)	
Obese (≥30)	393 (28.6)	193 (28.1)	200 (29.1)	
Heart rate, beats/min				
Low (<60)	92 (6.7)	28 (4.1)	64 (9.3)	<.001
Normal (60-100)	952 (69.3)	420 (61.1)	532 (77.4)	
High (>100)	330 (24)	239 (34.8)	91 (13.2)	
Systolic blood pressure, mm Hg				
Low (<90)	51 (3.7)	35 (5.1)	16 (2.3)	.05
Normal (90-120)	515 (37.5)	248 (36.1)	267 (38.9)	
Borderline (121-139)	409 (29.8)	206 (30.0)	203 (29.5)	
High (>139)	399 (29)	198 (28.8)	201 (29.3)	
Hypoxia (<90%)	111 (8.1)	68 (9.9)	43 (6.3)	.01
Creatinine, mg/dL				
Normal (0 to <1.2)	539 (39.2)	234 (34.1)	305 (44.4)	<.001
Elevated (1.2-2.0)	437 (31.8)	211 (30.7)	226 (32.9)	
Severely elevated (>2.0)	398 (29)	242 (35.2)	156 (22.7)	
Serum urea nitrogen, mg/dL				
Normal (0 to <17)	360 (26.2)	138 (20.1)	222 (32.3)	<.001
Elevated (17-25)	255 (18.6)	113 (16.4)	142 (20.7)	
Severely elevated (>25)	759 (55.2)	436 (63.5)	323 (47.0)	
Potassium, mEq/L				
Low (1.0 to <3.5)	326 (23.7)	182 (26.5)	144 (21.0)	.05
Normal (3.5-6.0)	1007 (73.3)	486 (70.7)	521 (75.8)	
High (>6.0)	41 (3.0)	19 (2.8)	22 (3.2)	
White blood cell count				
Low (0/μL to <2000/μL)	33 (2.4)	15 (2.2)	18 (2.6)	<.001
Normal (2000/μL to 10 000/μL)	581 (42.3)	223 (32.5)	358 (52.1)	
Elevated (>10 000/μL to <14 000/μL)	311 (22.6)	151 (22.0)	160 (23.3)	
Severely elevated (≥14 000/μL)	449 (32.7)	298 (43.4)	151 (22.0)	
Hemoglobin, g/dL				
Severely reduced (<8)	146 (10.6)	80 (11.6)	66 (9.6)	<.001
Moderately reduced (8 to <11)	635 (46.2)	393 (57.2)	242 (35.2)	
Mildly reduced (11 to <13 for men and 11 to <12 for women)	368 (26.8)	150 (21.8)	218 (31.7)	
Normal (≥13 for men and ≥12 for women)	225 (16.4)	64 (9.3)	161 (23.4)	
Sodium, mEq/L				
Low (<135)	403 (29.3)	219 (31.9)	184 (26.8)	<.001
Normal (135-145)	859 (62.5)	393 (57.2)	466 (67.8)	
High (>145)	112 (8.2)	75 (10.9)	37 (5.4)	

SI conversion factors: To convert creatinine to μmol/L, multiply by 88.4; serum urea nitrogen to mmol/L, multiply by 0.357; potassium to mmol/L, multiply by 1.0; white blood cell count to ×10^9^/L, multiply by 0.001; hemoglobin to g/L, multiply by 10.0; sodium to mmol/L, multiply by 1.0.

^a^ Calculated as weight in kilograms divided by height in meters squared.

The sequential model development for the identification of risk factors associated with in-hospital AMI is shown in eTables 1 to 3 in the Supplement. In case-control studies, the calculated exposure odds ratio approximates the disease odds ratio when the disease (in-hospital AMI) is sufficiently rare. From the final model, variables associated with an increased risk of in-hospital AMI included being married, a history of coronary artery disease, prior myocardial infarction, peripheral vascular disease, elevated heart rate, elevated serum urea nitrogen level, reduced hemoglobin level, and elevated white blood cell count (Table 2). Candidate variables that were associated with a lower risk of in-hospital AMI included low heart rate, atrial fibrillation, history of anemia, and depression.

**Table 2.** Independent Risk Factors Associated With In-Hospital Acute Myocardial Infarction

Variable	Odds Ratio (95% CI)^a^	*P* Value^b^
Married	1.8 (1.3-2.4)	<.001
Bed type		
Other vs medical bed	1.9 (1.0-3.4)	<.001
Intensive care unit vs medical bed	2.9 (1.9-4.4)	
Atrial fibrillation	0.6 (0.4-0.9)	.02
History of anemia	0.5 (0.3-0.7)	<.001
Coronary artery disease	1.9 (1.4-2.7)	<.001
Depression	0.7 (0.5-0.9)	.02
Prior myocardial infarction	2.0 (1.3-3.1)	.001
Peripheral vascular disease	2.0 (1.4-3.0)	.001
Heart rate		
Low vs normal	0.5 (0.3-0.9)	<.001
High vs normal	3.6 (2.5-5.3)	
Serum urea nitrogen		
Elevated vs normal	1.5 (1.0-2.3)	.01
Severely elevated vs normal	1.7 (1.2-2.4)	
Hemoglobin		
Reduced vs normal		<.001
Severely	2.8 (1.5-5.3)	
Moderately	4.4 (2.8-7.0)	
Mildly	1.5 (0.9-2.5)	
White blood cell count		
Low vs normal	0.7 (0.2-1.9)	<.001
Elevated vs normal	1.3 (0.9-2.0)	
Severely elevated vs normal	2.2 (1.6-3.1)	

^a^ The model included all covariates listed above as identified from sequential model builds (see eTables 1-3 in the Supplement).

^b^ *P* value for type 3 test of significance.

**Figure 2.**
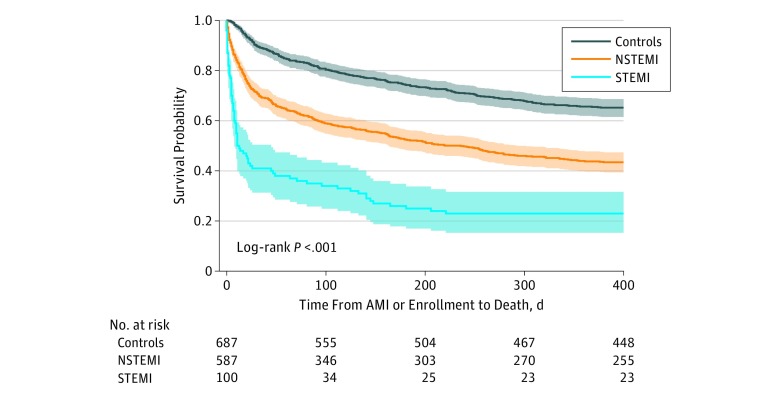
Kaplan-Meier Survival Curves for the Matched Cohort with In-Hospital Acute Myocardial Infarction (AMI) Cases Stratified by ST-Segment Elevation Myocardial Infarction (STEMI) and Non–ST-Segment Elevation (NSTEMI) The shaded areas show 95% confidence intervals.

In-hospital mortality among all eligible patients with in-hospital AMI was 25.3% and increased to 32.3% at 30 days and 58.4% at 1 year. In analyses of the matched cohort, cases experienced significantly higher mortality in-hospital than controls (26.4% vs 4.2%), at 30 days (33.0% vs 10.0%), and at 1 year (59.2% vs 34.4%), and risk-adjusted mortality was significantly higher for cases at all points (Table 3). Kaplan-Meier survival curves with cases stratified by STEMI vs NSTEMI demonstrated lowest survival following in-hospital STEMI (Figure 2). All-cause readmission within 1 year was 54.4% among case patients and did not significantly differ from the control group in unadjusted or adjusted comparisons (Table 3).

**Table 3.** Mortality and 1-Year Readmission for In-Hospital AMI Cases and Controls

Outcome	No. (%)				OR (95% CI)	
	Total	Cases	Controls	*P* Value	Unadjusted	Adjusted^a^
Mortality						
No.	1374	687	87			
In-hospital	210 (15.3)	181 (26.4)	29 (4.2)	<.001	8.12 (5.39-12.22)	6.67 (4.15-10.70)
30 d	296 (21.5)	227 (33.0)	69 (10.0)	<.001	4.42 (3.29-5.94)	4.01 (2.81-5.73)
1 y	643 (46.8)	407 (59.2)	236 (34.4)	<.001	2.78 (2.23-3.46)	2.41 (1.83-3.18)
Readmission						
No.	1164	506	658			
All cause	620 (53.3)	275 (54.4)	345 (52.4)	.52	1.08 (0.86-1.36)	0.97 (0.74-1.28)
Readmission for AMI	35 (3.0)	27 (5.3)	8 (1.2)	<.001	4.48 (2.06-10.17)	NA^b^

Abbreviations: AMI, acute myocardial infarction; NA, not applicable; OR, odds ratio.

^a^ Adjusted for all covariates in Table 1 using multivariable logistic regression models.

^b^ Not calculated because of small number of outcomes.

## Discussion

In this study from the VA health care system, we determined the incidence, risk factors, and outcomes associated with in-hospital AMI. A total of 5556 patients had in-hospital AMI during the study period, equating to a prevalence of 4.27 AMI events per 1000 admissions to VA hospitals. Risk factors associated with an increased risk of in-hospital AMI included a history of atherosclerotic disease and cardiovascular risk factors in addition to markers of physiological stress (eg, elevated heart rate, low hemoglobin, and elevated white blood cell count). While contemporary studies suggest the mortality for AMI that begins outside the hospital is approximately 13% at 30 days^15^ and 25% at 1 year,^16^ we observed mortality for in-hospital AMI that exceeded 25% at 30 days and approached 60% at 1 year. This study highlights the importance of in-hospital AMI as a common and high-risk clinical condition among hospitalized patients.

Compared with other in-hospital acquired conditions, in-hospital AMI occurs frequently. For example, studies of in-hospital cardiac arrest in VA populations have estimated event rates of 4 per 1000 hospital admissions.^17^ Prior research has sought to inform the optimal treatment of patients who experience in-hospital cardiac arrest.^18,19,20,21^ In addition, significant clinical resources are dedicated to in-hospital cardiac arrest, with estimates exceeding $300 million nationally to equip, train, and accredit clinicians and hospitals in resuscitation care.^22,23^ This attention to in-hospital cardiac arrest may be contributing to improvements in survival of this condition.^24,25,26^ Similar emphasis on research to understand the modifiable risks of in-hospital AMI and optimal treatment are lacking, despite a similar prevalence and poor long-term survival outcome of in-hospital AMI.

Decades of research have helped to clarify the risk factors associated with atherosclerosis and AMI onset out of the hospital.^27,28,29,30,31^ The present study suggests a history of atherosclerosis and risk factors for atherosclerosis contribute to the risk of in-hospital AMI, potentially reflecting a common pathway of spontaneous coronary plaque disruption and intraluminal thrombus formation (ie, type 1 MI by universal classification schema)^32^ for some in-hospital AMI. The present study also highlights the potential for physiological disturbances, such as severely low hemoglobin level, elevated heart rate, elevated serum urea nitrogen level, and elevated white blood count, to contribute to in-hospital AMI risk. This may reflect AMI events in the setting of myocardial injury resulting from an imbalance of myocardial oxygen supply relative to demand that is unrelated to coronary plaque disruption. These non–plaque disruption MIs, classified as type 2 MIs in current typology,^32^ have been associated with anemia, sepsis, and arrhythmia in prior studies of patients presenting with MI.^33,34,35^ The present study expands on this prior work by comparing with a non-AMI control group and identifying risk factors among patients with AMI that occurs during hospitalization. As the differentiation of type 1 MI (acute plaque disruption) and type 2 MI (myocardial oxygen supply demand mismatch) is challenging and often uncertain even at the point of care,^36^ no formal attempt was made in the present study to differentiate between these types of events from medical record review. Furthermore, the revision of MI classification to include type 2 MIs was first published in 2007,^14^ during the period under study.

Of the covariates associated with lower risk of in-hospital AMI, lower heart rate and history of anemia are worth particular mention. Lower heart rate may reflect homeostasis and further confirms the potential for physiological perturbation to contribute to in-hospital AMI risk. Medical record review of history of anemia was not further categorized into patients with persistent (chronic) anemia or those with resolved anemia. As such, the association between reduced hemoglobin level and in-hospital AMI risk may be more reflective of acute change rather than chronically low hemoglobin levels.

The nearly 60% mortality at 1 year following in-hospital AMI is striking. In comparison, mortality at 1 year after in-hospital cardiac arrest is 85%^26^ and the 1 year mortality for bronchus and lung cancer is approximately 50% in US surveillance programs.^37^ Although significant work remains to determine the extent to which in-hospital AMI outcomes can be modified by addressing cardiovascular risk and concurrent illness, these findings should prompt clinicians to recognize patients with in-hospital AMI as having a high mortality risk. Importantly, our study excluded patients with incidentally elevated troponin levels in the absence of concurrent signs and symptoms of myocardial ischemia. As such, our findings pertain to patients with clinical evidence of myocardial ischemia, as determined by symptoms and/or electrocardiographic changes in addition to a typical increase and decrease in cardiac enzymes.

Our study has several strengths, including the use of a complete AMI cohort to estimate the incidence of in-hospital AMI events and a nested case-control design to allow identification of in-hospital AMI risk factors and outcomes as compared with hospitalized non-AMI controls and eliminate selection bias, which is a risk of non-nested case-control design. The use of trained abstractors to extract data from medical records provided more complete and reliable information than complete reliance on computerized databases.

### Limitations

Limitations of our study include the VA health care setting, which reflects an older male population and may limit generalizability. Furthermore, our case-control study was restricted to patients aged 50 years and older with in-hospital AMI more than 24 hours after admission and excluded postsurgical patients, which also limits generalizability of our findings to medical admissions in older patients. It is worth noting that among the 1213 potential in-hospital AMI cases, only 26 (2.1%) were excluded from detailed medical record review for surgery in the preceding 72 hours. Second, case identification leveraged a quality-monitoring program that captures all hospitalized patients with AMI on the basis of *ICD-9* codes. Although the sensitivity (94%) and specificity (99%) of *ICD-9* codes for identification of patients with a discharge diagnosis of AMI is established,^38^ it is unclear whether these characteristics apply to the identification of in-hospital AMI. For the present study, issues of specificity were addressed through medical record review and case validation, but we cannot exclude the possibility of underdiagnosis of in-hospital AMI on the basis of *ICD-9* codes. Third, the effort required to complete medical record review along with unanticipated regulatory delays related to investigator transitions between institutions resulted in a delay between data acquisition and final analysis. However, this effort allowed for the completion of a case-control design that offered insights not provided from prior studies of in-hospital AMI, and the findings remain clinically relevant despite this latency. Fourth, although we attempted to obtain details on candidate risk factors as proximal to the event as possible, vital signs and laboratory values were often not available without evaluating at least 24 hours prior to the event. Fifth, our analyses of incidence and risk factors did not differentiate between STEMI and NSTEMI as our sample size was insufficient for stratified analyses. Furthermore, we did not attempt to differentiate between type 1 (acute plaque rupture) and type 2 (supply demand mismatch) because the differentiation between these events is often uncertain, even with access to the patient. Sixth, our incidence determination and subsequent nested case identification was dependent on *ICD-9* codes with uncertain sensitivity and specificity characteristics for events occurring in-hospital. Regardless, our study highlights the incidence, risk factors, and poor outcomes of patients who experience in-hospital AMI.

## Conclusions

In-hospital AMI is not infrequent, occurring in 4.27 of 1000 hospital admissions. From matched case-control design, factors associated with an increased risk of in-hospital AMI included history of atherosclerosis, traditional atherosclerotic risk factors, and markers of physiological stress. Outcomes following in-hospital AMI are poor, with nearly 60% mortality at 1 year after the event. Additional research to define risk reduction and optimal treatment strategies of in-hospital AMI are needed to address this common and high-risk condition.

## Notes

Supplement. **eTable 1.** Model Development for Risk-Factors of In-Hospital Myocardial Infarction **eTable 2.** Predictors and Outcomes of In-Hospital Acute Myocardial Infarction (IHAMI) Concordance of Data Abstraction—Phase 2 Preliminary Results **eTable 3.** Predictors and Outcomes of In-Hospital Acute Myocardial Infarction (IHAMI) Concordance of Data Abstraction—Preliminary Phase 3 Results
