# Supplementary material for: Incidence, Risk Factors, and Outcomes Associated With In-Hospital Acute Myocardial Infarction
Source: JAMA Netw Open. 2019 Jan 18;2(1):e187348. doi: 10.1001/jamanetworkopen.2018.7348 (PMC6484558; doi:10.1001/jamanetworkopen.2018.7348)
Supplement: Supplement. — eTable 1. Model Development for Risk-Factors of In-Hospital Myocardial Infarction eTable 2. Predictors and Outcomes of In-Hospital Acute Myocardial Infarction (IHAMI) Concordance of Data Abstraction—Phase 2 Preliminary Results eTable 3. Predictors and Outcomes of In-Hospital Acute Myocardial Infarction (IHAMI) Concordance of Data Abstraction—Preliminary Phase 3 Results [file jamanetwopen-2-e187348-s001.pdf]

Supplementary Online Content

Bradley SM, Borgerding JA, Wood GB, Maynard C, Fihn SD. Incidence, risk factors, and outcomes associated with in-hospital acute myocardial infarction. *JAMA Netw Open*. 2019;2(1):e187348. doi:10.1001/jamanetworkopen.2018.7348

eTable 1. Model Development for Risk-Factors of In-Hospital Myocardial Infarction

eTable 2. Predictors and Outcomes of In-Hospital Acute Myocardial Infarction (IHAMI) Concordance of Data Abstraction—Phase 2 Preliminary Results

eTable 3. Predictors and Outcomes of In-Hospital Acute Myocardial Infarction (IHAMI) Concordance of Data Abstraction—Preliminary Phase 3 Results

This supplementary material has been provided by the authors to give readers additional information about their work.

© 2019 Bradley SM et al. *JAMA Network Open.*

eTable 1. Model Development for Risk-Factors of In-Hospital Myocardial Infarction

Candidate Variables	Model 1: Demographics, bed type, and past history	Model 1a: Model 1 + acute conditions	Model 2: Model 1 + vitals	Model 3: Model 1 + labs	Model 4: Significant covariates
Gender	0.16	0.13	0.18	0.13	
Married	0.03 *	0.03 *	0.007 *	0.005 *	<0.001 *
Bed type	<0.001 *	<0.001 *	<0.001 *	<0.001 *	<0.001 *
White	0.39	0.4	0.33	0.46	
Atrial fibrillation	0.006 *	0.005 *	0.004 *	0.03 *	0.02 *
Alcohol dependence/abuse	0.19	0.16	0.74	0.39	
Anemia	0.05 *	0.06 *	0.06 *	<0.001 *	<0.001 *
Obstructive sleep apnea	0.55	0.49	0.65	0.8	
Prior coronary artery bypass graft	0.38	0.38	0.5	0.61	
Coronary artery disease	0.07 *	0.06 *	0.05 *	0.03 *	<0.001 *
Heart failure	0.42	0.54	0.26	0.28	
Chronic kidney disease	0.02 *	0.02 *	0.09 *	0.63	NS in final model
Chronic obstructive pulmonary disease	0.84	0.91	0.85	0.34	
Cerebrovascular disease	0.03 *	0.03 *	0.04 *	0.16	NS in final model
Dementia	0.19	0.2	0.17	0.12	
Depression	0.05 *	0.05 *	0.03 *	0.04 *	0.02 *
Diabetes	0.58	0.49	0.6	0.8	
Hypertension	0.57	0.56	0.6	0.46	
Hyperlipidemia	0.98	0.97	0.93	0.86	
Liver disease	0.2	0.22	0.31	0.36	
Malignant neoplasm	0.75	0.89	0.47	0.35	
Prior myocardial infaction	<0.001 *	<0.001 *	0.001 *	<0.001 *	0.001 *
Prior percutaneous coronary intervention	0.24	0.26	0.22	0.33	
Post-traumatic stress disorder	0.88	0.91	0.84	0.92	

© 2019 Bradley SM et al. *JAMA Network Open.*

Candidate Variables	Model 1: Demographics, bed type, and past history	Model 1a: Model 1 + acute conditions	Model 2: Model 1 + vitals	Model 3: Model 1 + labs	Model 4: Significant covariates
Peripheral vascular disease	<0.001 *	<0.001 *	<0.001 *	0.002 *	0.001 *
Tobacco use	0.69	0.68	0.63	0.27	
Coagulopathy		0.49			
Fluid/electrolyte disorder		0.39			
Gastrointestinal bleed		0.53			
Hypoxia		0.24			
Body mass index			0.33		
Systolic blood pressure			0.14		
Heart rate			<0.001 *		<0.001 *
Blood urea nitrogen				0.09 *	0.01 *
Creatinine				0.88	
Hemoglobin				<0.001 *	<0.001 *
Potassium				0.57	
Sodium				0.47	
White blood cell count				<0.001 *	<0.001 *

* Achieved threshold for inclusion in Model 4 (p<0.10)

© 2019 Bradley SM et al. *JAMA Network Open.*

eTable 2. Predictors and Outcomes of In-Hospital Acute Myocardial Infarction (IHAMI) Concordance of Data Abstraction – Phase 2 PRELIMINARY Results

	# Records Entered				
**Dataset**	**Abstr 1**	**Abstr 2**	**Abstr 3**	**Variable**	**% Agreement**
**Demographics**	77	77	77	**Gender** *(M, F)*	86% (66/77)
				**Ethnicity** *(AA, C)*	84% (65/77)
				**Marital Status** *(D, M, N, S, W)*	84% (65/77)
**Hospital Details**	174	168	165	**Admission Date**	82% (154/188)
				➢ **Discharge Date** [a]	94% (144/154)
**Discharge Diagnoses**	723	673	743	**Diagnosis**	80% (633/787)
**Follow-up**	432	435	597	**Follow-up Date**	51% (353/689)
				➢ **Follow-up Type** [a] *(APPT_CARDIOLOGY, APPT_PC, APPT_SURGEON, HOMECARE, TELEHEALTH, TEST_STRESS, etc.)*	89% (315/353)
**In-hospital Meds**	1495	1521	1630	**Med Name & Timing** *(BEFORE, DISCHARGE, AFTER)*	76% (1342/1764)
				➢ **Med Route** [a] *(INH, INJ, IV, MOUTH, NEB, SL, TOP)*	92% (1239/1342)
**Labs**	1892	1847	1953	**Lab Name & Date/Time**	64% (1478/2317)
**Vitals**	526	548	618	**Vital Type and Date/Time**	53% (396/744)

[a] For these variables, the denominator used to calculate the % agreement is based on the number of records that matched across abstractors according to the merging variable(s).

© 2019 Bradley SM et al. *JAMA Network Open.*

eTable 3. Predictors and Outcomes of In-Hospital Acute Myocardial Infarction (IHAMI) Concordance of Data Abstraction – Preliminary Phase 3 Results

	# Records			
**Dataset**	**Abstr**	**Abstr**	**Variable**	**% Agreement**
**Hospital Events**	446	360	All Events *(e.g., Dyspnea, Angina,*	74% (348/472)
			All Events & Dates	56% (262/472)
			All Events & When *(Event occurred before/on/after enrollment date)*	64% (301/472)
	68	58	Dyspnea/Shortness of breath<br>- SOB & When	94% (66/70)<br>80% (56/70)
	31	22	Angina<br>- Angina & When	71% (22/31)<br>65% (20/31)
	29	21	Hypotension<br>- Hypotension & When	70% (21/30)<br>57% (17/30)
**Procedures**	279	260	All Procedures *(e.g., EKG, Cardiology,*	84% (251/299)
			All Procedures & Dates	66% (196/299)
			All Procedures & End Dates	67% (195/299)
			All Procedures & Physician Deferred	81% (241/299)
			All Procedures & Patient Declined	83% (248/299)
	51	45	EKG done<br>- EKG & Date	85% (44/52)<br>54% (28/52)
	43	40	Cardiology done<br>- Cardiology & Date	93% (40/43)<br>86% (37/43)
	23<br>37	22<br>37	Transfusion done<br>- Transfusion & Date	88% (21/24)<br>48% (21/44)
	36	34	Echo done<br>- Echo & Date	84% (32/38)<br>68% (26/38)
	26<br>32	27<br>31	Cath/PCI done<br>- Cath/PCI & Date	96% (26/27)<br>73% (24/33)
	17	18	Stress test done<br>- Stress test & Date	89% (17/19)<br>84% (16/19)

© 2019 Bradley SM et al. *JAMA Network Open.*

	# Records Entered			
**Dataset**	**Abstr**	**Abstr**	**Variable**	**% Agreement**
**Cath Lab Details**	15	15	Type *(Cath, PCI, Both)*	100% (15/15)
			Type & Bare metal stent	93% (14/15)
			Type & Drug eluting stent	93% (14/15)
			Type & Distal protection	100% (15/15)
			Type & Thrombus removal	100% (15/15)
	1	0	Type & Complications	(14/15 w/ no compl)
**EKG Details**	73	71	Type *(e.g., Ischemic changes, Normal, ST-depression, T- wave changes)*	57% (52/92)
**Echo Details**	28	26	Source *(Hospital floor, Cath, Prev Hx)*	73% (22/30)
			EF Percent *(Normal≥55%, Mild 45-54%, Moderate 30- 44%, Severe<30%)*	60% (18/30)
			Wall Motion *(Normal, Abnormal)*	63% (19/30)
**Stress Test Details**	6	2	Type *(Nuclear, EKG, Echo)*	Database would not allow partial
			Method *(Pharmacologic, Maximal, Sub-*	-
			Result *(Negative, Positive, Equivocal,*	-
			Risk *(Low, Medium, High)*	-

© 2019 Bradley SM et al. *JAMA Network Open.*
